# French Consumption of Methylphenidate in Primary Care From 2016 to 2023, Impact of Prescribing Policy Changes—A Time‐Series Analysis

**DOI:** 10.1002/pds.70424

**Published:** 2026-07-01

**Authors:** Claire Saudreau, Vincent Tarazona, David De Bandt

**Affiliations:** ^1^ Department of General Medicine, UFR de la santé Simone Veil Université de Versailles Saint‐Quentin Montigny le Bretonneux France; ^2^ Equipe soins primaires et prévention (CESP) INSERM U 1018, Université Paris‐Saclay, UVSQ, Université Paris‐Sud Villejuif France

**Keywords:** ADHD, health expenditure, health policies, methylphenidate, pharmacoepidemiology

## Abstract

**Purpose:**

In France, methylphenidate, mainly used in the treatment of ADHD, has been subject to prescription restrictions that were relaxed at the end of 2021. This study analyses trends in methylphenidate consumption in France and examines changes following the modification to prescribing rules in 2021.

**Methods:**

This ecological study was based on data from the Medic'AM database, which records reimbursed outpatient drug dispensation in France from January 2016 to December 2023. Methylphenidate sales were expressed as defined daily dose per thousand inhabitants per day (DDD/TID) and expenditure as euros per thousand inhabitants. Time‐series analyses were conducted to assess changes in methylphenidate sales and associated expenditure following modifications to prescribing arrangements in September and November 2021.

**Results:**

Methylphenidate consumption rose from 0.607 DDD/TID per month in 2016 to 1.457 DDD/TID in 2023, an increase of 84%. Associated expenditure followed a similar upward trend. A more pronounced increase in methylphenidate sales was observed after the end of 2021.

**Conclusion:**

The study shows a clear increase in methylphenidate sales after 2021, coinciding with changes in prescribing regulations. Given the ecological design, this temporal association cannot be interpreted as causal. The observed trends likely reflect multiple factors, including regulatory changes, increased recognition of ADHD, and evolving clinical practices. These findings highlight how changes in prescribing policies may be associated with variation in healthcare utilization and expenditure.

## Key Points

- Methylphenidate use in France rose 84% from 2016 to 2023, reaching 1.457 DDD/TID.
- Prescription rule relaxations in 2021 are associated with a marked rise in consumption.
- Sales growth mirrored rising public health insurance expenditure trends.
- Time‐series analyses showed significant post‐2021 increases in use and expenditure.
- Expanded prescribing access improved treatment availability, boosting overall demand.

## Plain Language Summary

In France, methylphenidate, a medication mainly used to treat attention‐deficit/hyperactivity disorder (ADHD), has been subject to strict prescribing rules, which were relaxed at the end of 2021. These changes allowed community‐based specialists to initiate treatment and extended access to adults. Using national health insurance data on outpatient drug dispensations from 2016 to 2023, this study examined trends in methylphenidate sales over time. Use increased by 84% during this period, along with a rise in related healthcare expenditure. A more marked increase was observed after the changes in prescribing rules in 2021. Because this study was based on aggregated data, it cannot determine whether these policy changes directly caused the increase. However, the findings suggest that regulatory changes, along with other factors such as improved recognition of ADHD, may have contributed to increased use of methylphenidate. Overall, this study shows that changes in prescribing rules may be associated with changes in medication use and healthcare spending.

## 1 Introduction

Attention‐deficit hyperactivity disorder prevalence, with or without hyperactivity (ADHD), is estimated at around 8% of children and 3% of adults, with high heterogeneity across the studies [1, 2, 3, 4]. The DSM‐5 describes ADHD as a chronic condition with a neurodevelopmental disorder that leaves patients with a functional handicap [5]. Clinical forms exist according to three main symptoms of varying intensity: attention deficit, hyperactivity, and impulsiveness. Early detection of ADHD is crucial to mitigate its worsening impact. Repercussions can be psychological with anxiety‐depressive disorders, academic with the risk of a drop in performance that can even lead to dropping out of school, and social with the risk of isolation. Despite its repercussions on patient well‐being, ADHD is difficult to diagnose, and over half of the patients suffering from ADHD are undiagnosed [6].

Methylphenidate has been approved by the Food and Drug Administration of the United States (FDA) for ADHD treatment since 1961 and by the French drug administration (ANSM) since October 1995 [7, 8]. It had also obtained a more marginal marketing authorization as a second‐line treatment for narcolepsy in 1999. In France, methylphenidate is listed as a narcotic and must be prescribed on a secure prescription for a maximum of 28 days. These prescriptions must be written on watermarked paper, with a serial number. They must be handwritten, spelled out in full, without abbreviations, and specifying the dispensing pharmacy. The prescribing doctor's signature and contact details must appear. Initial and annual prescriptions must be issued by hospital specialists, notably pediatricians, neurologists, or psychiatrists. In the meantime, any doctor can renew the prescription [9].

In 2014, in France, only 48 895 children were prescribed methylphenidate at least once [9, 10]. The rate of patients treated with methylphenidate in France remains lower than in other European countries and North America, leading to fears that there will be a greater number of undiagnosed and untreated patients [9, 11]. Given the importance of early treatment for patients suffering from ADHD, on September 13, 2021, the initial prescription and annual renewal of methylphenidate were authorized for pediatricians, neurologists, and psychiatrists working in the city sector [12]. Moreover, on November 17, 2021, the French health authorities gave a favorable opinion on the use of methylphenidate for the treatment of ADHD in adults [13]. The broadening of reimbursement by the French health insurance system for adults treated with methylphenidate was authorized by a decree dated June 29, 2023 [14].

These decrees reflect an effort to improve access to methylphenidate for patients suffering from ADHD; however, no study has yet examined the impact of these reforms on drug consumption.

The primary objective of this study was to assess methylphenidate consumption in France from January 2016 to December 2023 in order to gain a better understanding of French consumption patterns. The secondary objectives were to gain a better understanding of the impact of healthcare policies on methylphenidate consumption in France and to determine changes in costs to the French public health insurance system. We hope that this study will help to understand the dynamics of changes in methylphenidate consumption in response to the extension of its prescribing rules.

## 2 Methods

### 2.1 Experimental Design and Database

This is an ecological study of the French population from January 2016 to December 2023. This study is not an interventional study on human beings. The databases used are anonymized and freely accessible on the internet. No ethical authorization was required.

The data were extracted from the Medic'AM database for the period from January 2016 to December 2023 [15]. This database compiles data on medication sales in community pharmacies that are reimbursed by the French public health insurance system. Data are reported as monthly sales by ATC5 classification, corresponding to the specific active substance, and by CIP13 code, which identifies each medicine according to its trade name, pharmaceutical form, dosage per unit, and number of units per package. Medic'AM also records the number of boxes sold each month and the associated monthly expenditure reimbursed by the French public health insurance system. In France, methylphenidate is dispensed by pharmacies upon presentation of a written prescription with a serial number and issued by a licensed physician. The French public health insurance system covers 65% of the cost of methylphenidate for patients insured under the French general scheme, which accounts for approximately 90% of the population [16]. Reimbursement may reach 100% for patients officially recognized as suffering from a long‐term condition.

Data on the French population census were extracted from the National Institute of Statistics and Economic Studies (INSEE) database from 2016 to 2023. This database records the number of inhabitants in France on January 1 of each year [17].

### 2.2 Variables of Interest

The primary endpoint was the mean monthly defined daily dose per thousand inhabitants per day (DDD/TID) sold in pharmacies in France. This measure is recommended by the World Health Organization (WHO) for studies of drug consumption and represents the daily dose indicated for the main indication of the compound [18]. All monthly data for the ATC5 class N06BA04 representing methylphenidate were extracted for the study period. In France, methylphenidate is the only medication with a marketing authorization and reimbursement for the treatment of ADHD in outpatient care during the study period. Other pharmacological options commonly used in other countries were not included in this analysis, as atomoxetine is not reimbursed and therefore not captured in the Medic'AM database, modafinil does not have an indication for ADHD, and other agents such as lisdexamfetamine and guanfacine were not marketed in France.

The monthly DDD/TID was calculated by multiplying the dose per unit by the number of units per box and by the number of boxes sold per month for each IPC13, divided by the DDD of methylphenidate, by the number of days in the month, and by the number of inhabitants on the first of January of the year, with a factor of 1.000. The DDD for methylphenidate is 30 mg/day according to the WHO [19].

$$\begin{array}{c} \text{Monthly}\;\mathrm{DDD}/\mathrm{TID}\\ \;=\frac{\text{Volume sold during the month}\times 1000\;\text{inhabitants}\;}{\mathrm{DDD}\times \text{total population}\times \text{number of days in the month}} \end{array}$$

Expenditures associated with DDD/TID were adjusted for inflation to December 31, 2023, and presented in euros per 1000 inhabitants per year [20].

### 2.3 Statistical Analysis

This study analyzed the impact of the extension of methylphenidate prescribing rules on methylphenidate sales from September 2021 onwards in a multistage process. The date of publication of the new methylphenidate prescribing authorizations meant that September 2021 was chosen as the breaking point for the analysis. An initial analysis of the data showed a stationary trend with autocorrelations and seasonality in methylphenidate sales over the periods January 2016 to September 2021 and September 2021 to December 2023 (Appendix I).

Data on methylphenidate consumption and expenditure were analyzed using several time series models, including Newey–West regression models, month‐adjusted models, Fourier transform models, and Autoregressive Integrated Moving Average (ARIMA) models with and without seasonal differencing, as well as ARIMA models without differencing. These models were compared to perform a sensitivity analysis and to identify the best‐performing predictive model (Appendices II and III).

ARIMA models without differencing were selected for the main analysis, as they demonstrated comparable robustness to ARIMA models with and without seasonal differencing, while accounting for seasonality and autocorrelation and remaining simpler to interpret. The data were analyzed according to monthly trends before, during, and after the September 2021 breaking point, along with 95% confidence intervals (95% CIs).

Predictive models of DDD/TID and associated expenditure from September 2021 to December 2023 were produced using data before September 2021. The difference in DDD/TID methylphenidate sales and associated expenditure after the breaking point between the predicted and observed models was calculated using Monte Carlo simulations with 1 million iterations. Excess methylphenidate sales and expenditure over the period from September 2021 to December 2023 were calculated and presented in total DDD/TID and total expenditure in euros per 1000 inhabitants using the 95% prediction interval (95% PI) and assuming a normal distribution of the predicted data. Prediction intervals were used instead of confidence intervals, as they reflect the uncertainty around individual predicted values rather than the mean estimate.

All analyses were performed on RStudio (version 20.12.2023+402).

## 3 Results

### 3.1 Descriptive Results

The French population increases progressively over the study period, rising from 66.602 million inhabitants on January 1, 2016, to 68.143 million on January 1, 2023. The evolution of the French population follows a linear trend (Appendix IV).

**FIGURE 1:**
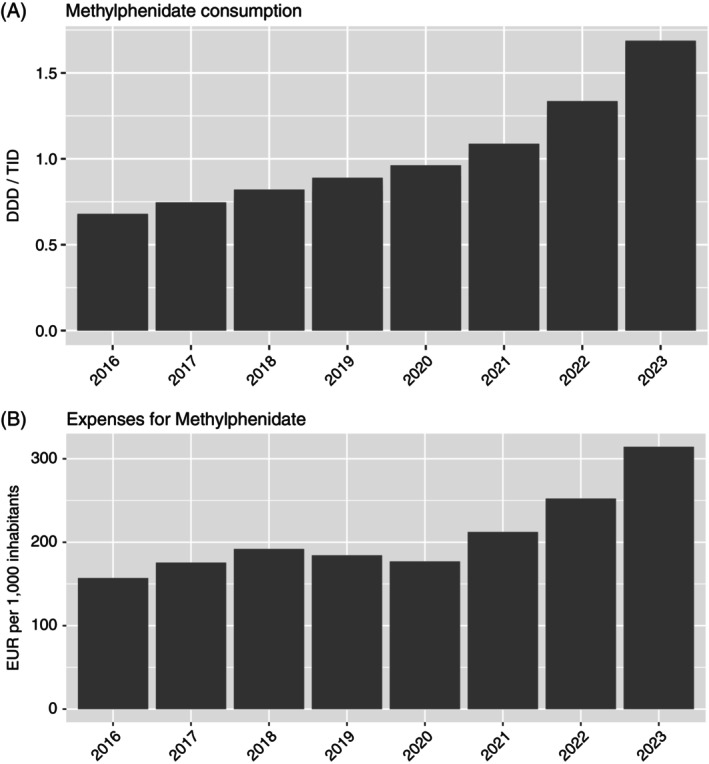
(A) Change in average monthly consumption of methylphenidate in DDD/TID between 2016 and 2023. (B) Change in euros per 1000 inhabitants per year linked to methylphenidate use between 2016 and 2023.

Methylphenidate sales do not follow a linear trend and increase from a monthly mean of 0.607 DDD/TID in 2016 to 0.791 DDD/TID in 2020, an increase of 30.313%. Sales of methylphenidate then increase more sharply, reaching 1.457 DDD/TID in 2023, an increase of 84.197% (Figure 1).

Expenditure per 1000 inhabitants on methylphenidate sales follows a nonlinear trend. This expenditure varies from €157.089 per 1000 inhabitants in 2016 to €191.908 in 2018, an increase of 22.165%. Mean monthly expenditure then falls to €177.004 in 2020, a decrease of 7.762%. Expenditure then rises sharply to €314.380 per 1000 inhabitants in 2023, an increase of 77.611% (Figure 1).

### 3.2 Impact of Changes in Prescribing Arrangements

September 2021 was chosen as the breaking point, the date of extension of methylphenidate prescribing rules. Segmented analyses show a significant increase in methylphenidate sales before the change point, with an average monthly coefficient of variation of 0.006 DD/TID (95% CI = 0.003; 0.008, *p* < 0.001). No significant change is observed at the breaking point. In contrast, methylphenidate sales increased in the period following the change point, with an average monthly coefficient of variation of 0.018 DDD/TID (95% CI = 0.012; 0.024, *p* < 0.001) compared with the period before September 2021 (Table 1).

**TABLE 1:** Segmented analysis of monthly methylphenidate consumption and expenditure between January 2016 and December 2023, with September 1, 2021, as the change point.

	Pre‐publication trend		Immediate level change		Change in trend		Excess
	estimate (95% CI)	*p*	estimate (95% CI)	*p*	estimate (95% CI)	*p*	estimate (95% PI)
DDD/TID	0.006 (0.003–0.008)	< 0.001	−0.031 (−0.100 to 0.038)	0.371	0.018 (0.012–0.024)	< 0.001	6.782 (5.961–7.603)
Euros per 1000 inhabitants	0.080 (0.016–0.145)	0.014	−0.682 (−2.181 to 0.816)	0.372	0.329 (0.184–0.474)	< 0.001	115.468 (96.763–134.175)

Abbreviations: 95% CI, 95% confidence interval; 95% PI, 95% prediction interval; DDD/TID, defined daily dose per thousand inhabitants a day; *p*, statistical value.

Expenditure on methylphenidate sales increased significantly between January 2016 and September 2021, with an average monthly coefficient of variation of €0.080 per 1000 inhabitants (95% CI = 0.016; 0.145, *p* = 0.014). There is no significant variation in expenditure associated with methylphenidate sales at the change point. Expenditure on methylphenidate sales increases more sharply after the change point, with an average monthly coefficient of variation of €0.329 per 1000 inhabitants (95% CI = 0.184; 0.474, *p* < 0.001) compared with the period before September 2021 (Table 1).

**FIGURE 2:**
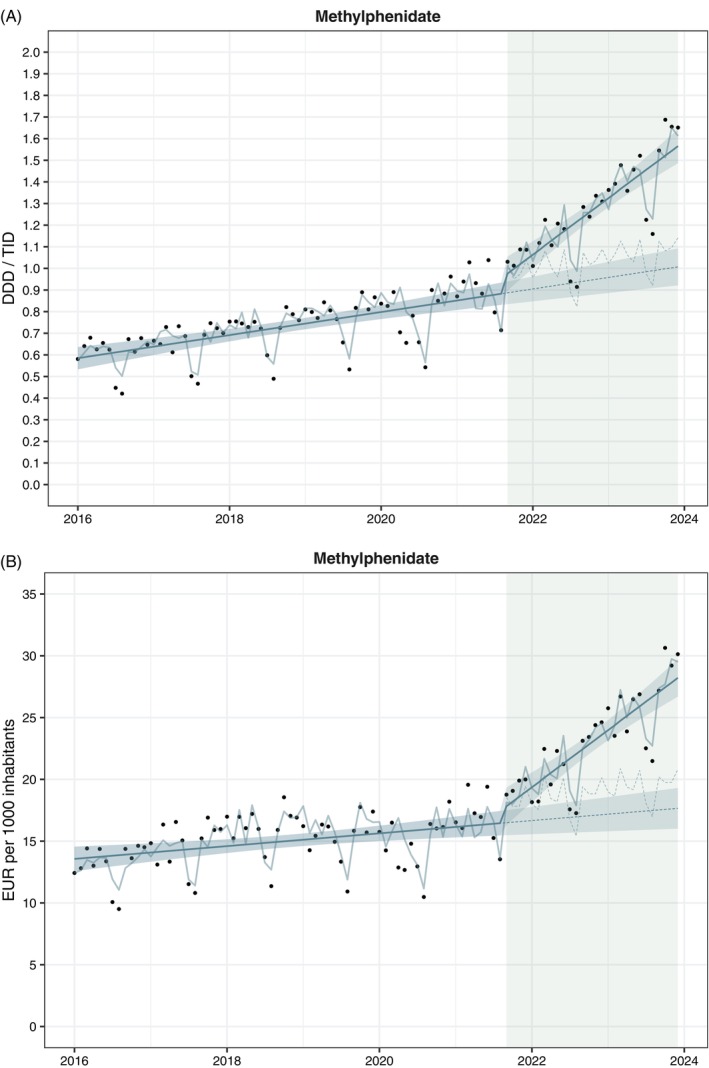
(A) Trend in monthly consumption of methylphenidate from January 2016 to December 2023 in DDD/TID. (B) Trend in monthly expenditure on methylphenidate consumption in euros per 1000 inhabitants from January 2016 to December 2023. Black dots: Observed data: the period before the change point on September 1, 2021 is highlighted in white; the period after the change point is highlighted in green; Green curve: Autoregressive model with actual observations; dotted curve: Model predicted from data before the change point; green line: Linear regression model of actual observations; and dotted line: Linear regression model of the predictive model.

The difference in methylphenidate sales between the predicted model and actual observations from September 2021 to December 2023 shows an excess of 6.781 DDD/TID (95% PI = 5.960; 7.603). The associated excess of expenditure represents €115.468 per 1000 inhabitants (95% PI = 96.763; 134.175) (Figure 2).

## 4 Discussion

### 4.1 Main Results

In France, consumption of methylphenidate increased gradually between 2016 and 2020, followed by a sharper rise after September 2021, coinciding with the extension of prescribing conditions to community‐based specialists and, shortly thereafter, to adult patients. While this study identifies a clear temporal association between the rise in methylphenidate sales observed from the end of 2021 and the implementation of the new prescribing regulations, causality cannot be inferred from this ecological design. The post‐2021 increase in methylphenidate sales corresponds to an estimated excess of 6.781 DDD/TID between September 2021 and December 2023 compared with model‐based predictions.

### 4.2 Comparison With Literature

Sales of methylphenidate increased progressively from January 2016 to September 2021 in France. These findings are consistent with observations reported in other European countries, Australia, Israel, and Turkey [21, 22, 23, 24]. The increase in methylphenidate sales may be related to a rise in ADHD diagnoses [25, 26, 27]. In France, the number of children diagnosed with ADHD increased by 96% between 2010 and 2019 [11]. However, the overall prevalence of ADHD worldwide does not appear to have increased [28, 29]. The increase in methylphenidate sales may therefore reflect the rise in awareness and recognition of ADHD. Children suspected of suffering from ADHD may be more easily identified and referred to healthcare professionals, leading to increased diagnosis and treatment [30]. Changes in diagnostic criteria may also have contributed to this trend. The transition from DSM‐IV to DSM‐5 broadened the definition of ADHD, particularly for adults, which may have increased the number of individuals eligible for diagnosis and treatment over time.

Another factor that may have contributed to increased methylphenidate sales is off‐label prescribing, with treatment initiation by general practitioners (GPs) despite official restrictions limiting initiation to hospital‐based specialists in neurology, pediatrics, and psychiatry. In 2017, one‐third of treatment initiations and half of renewals occurred outside the hospital setting, primarily by GPs [9]. This likely reflects challenges related to access to hospital‐based specialists and unevenness of the medical workforce [31, 32, 33]. Limited access to specialists combined with patient demand may have led to a shift in prescribing practices toward community‐based care. To date, no study has specifically examined the relationship between access to hospital psychiatrists, pediatricians, and neurologists and the number of methylphenidate treatment initiations in general practice.

Methylphenidate consumption may also have increased due to difficulties in accessing healthcare professionals who provide non‐pharmacological interventions for ADHD, which are recommended as first‐line treatments. These professionals often offer services that are costly and insufficiently reimbursed by health insurance systems [34]. Limited access to such nonmedical professionals in the management of ADHD may have contributed to an increased reliance on methylphenidate as a first‐line therapeutic option. Future research could usefully explore the association between the initiation of methylphenidate treatment and patients' access to nonmedical primary care providers involved in ADHD management, such as psychologists, psychomotor therapists, speech and language therapists, occupational therapists, and school support staff.

Other factors may also have contributed to the increase in methylphenidate sales, such as publications showing the effectiveness of these treatments in ADHD or the influence of pharmaceutical industry marketing campaigns. But these effects are more difficult to demonstrate [35, 36].

Following the extension of methylphenidate prescribing rights to community‐based specialists and adult patients in September and November 2021, an increase in methylphenidate sales was observed. These regulatory changes have expanded both the number of physicians authorized to initiate methylphenidate treatment and the population eligible to receive it. Previous studies have demonstrated that greater availability of prescribing physicians and an expanded patient population are associated with increased pharmaceutical sales [37, 38]. However, given the ecological design of this study, it is not possible to determine the respective contribution of these regulatory changes to the observed increase in methylphenidate consumption. The rate of off‐label prescribing should fall with these new prescribing arrangements. Further research is warranted to determine whether the number of first‐time off‐label methylphenidate prescriptions has indeed decreased since these reforms.

It also remains difficult to determine the respective roles of expanded prescriber eligibility and the extension of treatment to adults. The absence of reimbursement for adult methylphenidate prescriptions until June 2023 may also have limited sales within this population. Moreover, uncertainties regarding the efficacy of methylphenidate in adults may have influenced prescribing practices. International data suggest that pharmacological treatment of ADHD is substantially less frequent in adults than in children and adolescents [39, 40, 41]. Changes in the age structure of the population may also have influenced overall trends. However, the Medic'AM database provides only aggregated data and does not include age information, preventing age‐stratified analyses or adjustment for demographic changes. Further research is needed to examine methylphenidate prescribing patterns and to characterize the patient populations receiving treatment in France.

Finally, the increases in methylphenidate sales observed after 2021 may partly reflect a catch‐up effect among patients who had previously remained untreated due to prescribing restrictions. The 2017 ANSM report had already suggested that ADHD may be underdiagnosed in France compared with other countries, particularly the United States [9, 11].

### 4.3 Strengths and Limitations of the Study

The main strengths of this study lie in the completeness and originality of its data. To our knowledge, it is the first study to examine the impact of public health policies on methylphenidate use. The data were derived exclusively from community dispensations, excluding hospital deliveries, which may have introduced a potential source of confounding bias. In France, community pharmacies supply the majority of routinely used medications, whereas hospital pharmacies primarily dispense drugs to inpatients, distribute exceptional‐use medications, or participate in specific clinical research protocols [42]. All statistical analyses were validated, and sensitivity assessments were performed for the predictive models to ensure the robustness of the findings.

However, this study has some limitations. First, its ecological design does not allow a causal relationship between the publication of the new prescription conditions at the end of 2021 and the observed increase in methylphenidate consumption. Second, because the data were anonymized and aggregated, it was not possible to determine the exact number of individuals treated with methylphenidate. In addition, no information on age was available, preventing stratification of methylphenidate use between children, adolescents, and adults. This is an important limitation, as treatment patterns differ substantially across age groups, and the relative contribution of adult ADHD treatment to the post‐2021 increase could not be directly assessed. The data also include methylphenidate dispensed as a second‐line treatment for narcolepsy. Although these prescriptions are likely marginal, they may lead to a slight overestimation of ADHD‐related sales. In addition, ongoing supply shortages of methylphenidate since February 2022 may have influenced the results. According to the TDA France website, these shortages have persisted and even worsened over time, which may have limited dispensing and led to an underestimation of actual demand [43]. Furthermore, pharmacy dispensing data may underestimate the number of prescriptions issued by physicians and may overestimate actual patient consumption. Finally, this study focused exclusively on methylphenidate, as it is the only medication with market authorization for ADHD in France. This limits the generalizability of our findings to countries where other pharmacological options, such as atomoxetine, modafinil, lisdexamfetamine, or guanfacine, are available and commonly used.

Regarding expenditure, price variations due to negotiations between the health insurance system and the pharmaceutical companies limit the interpretation of temporal trends. As negotiation data were not available, it was not possible to fully analyze the determinants of expenditure changes.

## 5 Conclusion

This study shows that the extension of prescribing conditions to community‐based specialists and adult patients was temporally associated with an increase in methylphenidate sales in France. These findings highlight how changes in prescribing regulation may influence drug sales patterns. Such policies should therefore be carefully designed and evaluated to ensure appropriate access to treatment while minimizing the risk of inappropriate use.

## Funding

The authors have nothing to report.

## Ethics Statement

The authors have nothing to report.

## Conflicts of Interest

The authors declare no conflicts of interest.

## Supporting information


**Annex IA.** Supporting Information.

**Annex IB.** Supporting Information.

**Appendix I.** Decomposition of time series. (A) Breakdown of monthly consumption of methylphenidate in DDD/TID. (B) Breakdown of monthly expenditure in euros per 1000 inhabitants.
**Appendix II**. Sensitivity analyses of the segmented models used to study monthly consumption of methylphenidate in DDD/TID from January 2016 to December 2023. 95% CI, 95% confidence interval; 95% PI, 95% prediction interval; AIC, Akaike information criterion; BIC, Bayesian information criterion; DDD/TID, defined daily dose per thousand inhabitants per day; p, statistical value; RMSE, root mean square error; RSQ, R‐squared.
**Appendix III**. Sensitivity analyses of the segmented models used to study monthly expenditure in euros per 1000 inhabitants linked to methylphenidate consumption from January 2016 to December 2023. 95% CI, 95% confidence interval; 95% PI, 95% prediction interval; AIC, Akaike information criterion; BIC, Bayesian information criterion; p, statistical value; RMSE, root mean square error; RSQ, R‐squared.
**Appendix IV**. Data with year aggregation.

## Data Availability

The data that support the findings of this study are openly available in datagouv at https://www.data.gouv.fr/datasets/medicam‐medicaments‐rembourses‐par‐lassurance‐maladie‐par‐type‐de‐prescripteur‐donnees‐interregimes.
