# Supplementary figures and images for: French Consumption of Methylphenidate in Primary Care From 2016 to 2023, Impact of Prescribing Policy Changes—A Time‐Series Analysis
Source: Pharmacoepidemiol Drug Saf. 2026 Jul 1;35(7):e70424. doi: 10.1002/pds.70424 (PMC13324226; doi:10.1002/pds.70424)

# Methylphenidate, decomposition of additive time series (DDD/TID)

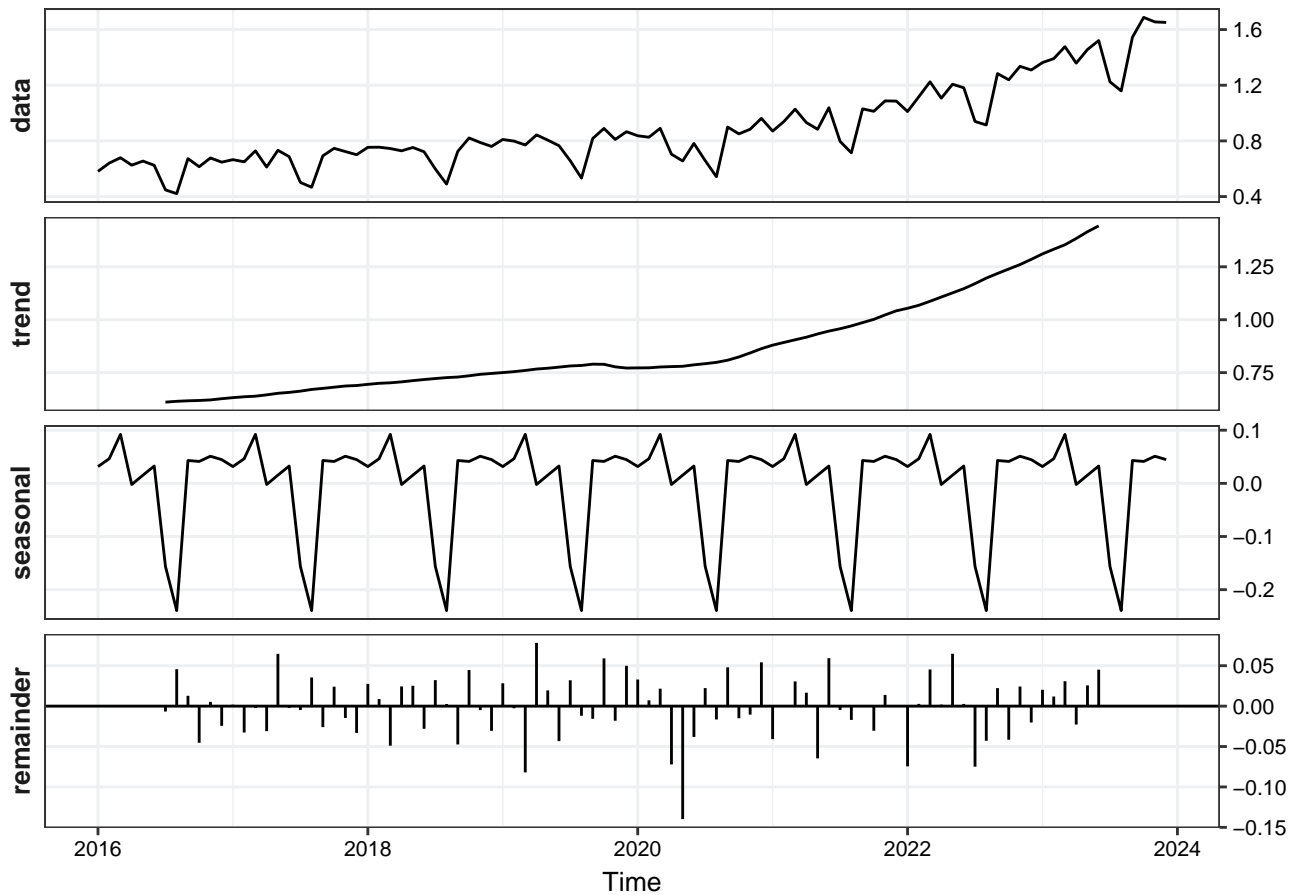

Supplement: Supplementary file 1 — Annex IA. Supporting Information. [file PDS-35-e70424-s001.pdf]

# Methylphenidate, decomposition of additive time series (EUR per 1000 inhabitants)

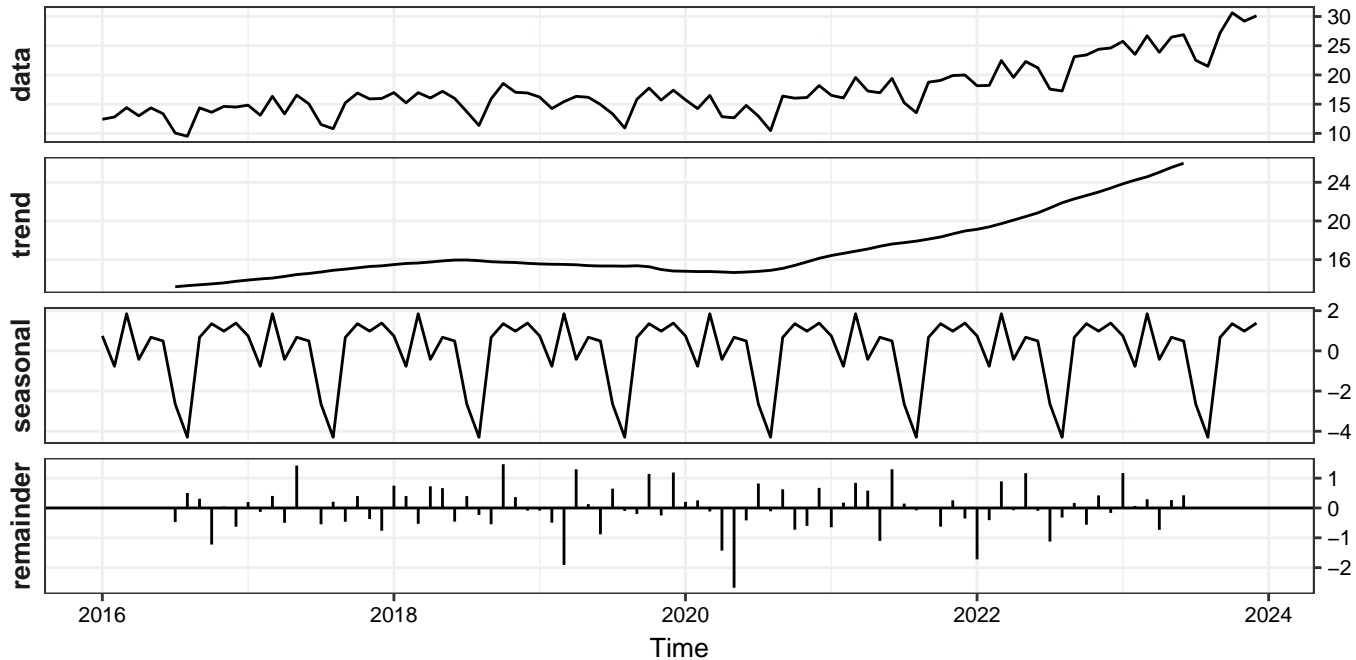

Supplement: Supplementary file 2 — Annex IB. Supporting Information. [file PDS-35-e70424-s003.pdf]
