# Supplementary material for: French Consumption of Methylphenidate in Primary Care From 2016 to 2023, Impact of Prescribing Policy Changes—A Time‐Series Analysis
Source: Pharmacoepidemiol Drug Saf. 2026 Jul 1;35(7):e70424. doi: 10.1002/pds.70424 (PMC13324226; doi:10.1002/pds.70424)
Supplement: Supplementary file 3 — Appendix I. Decomposition of time series. (A) Breakdown of monthly consumption of methylphenidate in DDD/TID. (B) Breakdown of monthly expenditure in euros per 1000 inhabitants. Appendix II. Sensitivity analyses of the segmented models used to study monthly consumption of methylphenidate in DDD/TID from January 2016 to December 2023. 95% CI, 95% confidence interval; 95% PI, 95% prediction interval; AIC, Akaike information criterion; BIC, Bayesian information criterion; DDD/TID, defined daily dose per thousand inhabitants per day; p, statistical value; RMSE, root mean square error; RSQ, R‐squared. Appendix III. Sensitivity analyses of the segmented models used to study monthly expenditure in euros per 1000 inhabitants linked to methylphenidate consumption from January 2016 to December 2023. 95% CI, 95% confidence interval; 95% PI, 95% prediction interval; AIC, Akaike information criterion; BIC, Bayesian information criterion; p, statistical value; RMSE, root mean square error; RSQ, R‐squared. Appendix IV. Data with year aggregation. [file PDS-35-e70424-s002.docx]

**French consumption of methylphenidate in primary care from 2016 to 2023, impact of prescribing policy changes, a time series analysis.**

# **Supplementary data**

### **Appendix I:** Decomposition of time series. A: breakdown of monthly consumption of methylphenidate in DDD/TID, B: breakdown of monthly expenditure in euros per 1,000 inhabitants.

| **DDD/TID** | **Trend (95%CI)** | **p** | **AIC** | **BIC** | **RMSE** | **RSQ** | **Adj-RSQ** | | **Excess of DDD/TID (95%PI)** |
| --- | --- | --- | --- | --- | --- | --- | --- | --- | --- |
| **Newey-West Standard Error Regression** | | | | | | | | | |
| Intercept | 0.580 (0.557 ; 0.604) | <0.001 | -148.862 | -136.040 | / | 0.869 | 0.864 | 9.061 (7.904 ; 10.217) | |
| Pre change trend | 0.004 (0.004 ; 0.005) | <0.001 |  |  |  |  |  |  | |
| Instant trend | 0.071 (0.003 ; 0.140) | 0.043 |  |  |  |  |  |  | |
| Post change trend | 0.017 (0.013 ; 0.021) | <0.001 |  |  |  |  |  |  | |
| **Regression with Month Adjustment** | | | | | | | | | |
| Intercept | -115.496 (-130.395 ; -100.597) | <0.001 | -284.780 | -243.751 | / | 0.975 | 0.970 | 8.305 (7.725 ; 8.885) | |
| Pre change trend | 0.058 (0.050 ; 0.065) | <0.001 |  |  |  |  |  |  | |
| Instant trend | 0.025 (-0.021 ; 0.071) | 0.285 |  |  |  |  |  |  | |
| Post change trend | 0.019 (0.016 ; 0.021) | <0.001 |  |  |  |  |  |  | |
| **Regression with Fourier Transform** | | | | | | | | | |
| Intercept | 0.574 (0.537 ; 0.612) | <0.001 | -209.870 | -186.791 | / | 0.936 | 0.931 | 8.558 (7.701 ; 9.416) | |
| Pre change trend | 0.005 (0.004 ; 0.006) | <0.001 |  |  |  |  |  |  | |
| Instant trend | 0.043 (-0.027 ; 0.112) | 0.229 |  |  |  |  |  |  | |
| Post change trend | 0.018 (0.014 ; 0.022) | <0.001 |  |  |  |  |  |  | |
| **ARIMA model with seasonal and non-seasonal differencing** | | | | | | | | | |
| ar1 | -0.439 (-1.472 ; 0.593) | 0.404 | -227.066 | -210.134 | 0.051 | / | / | 4.846 (3.518 ; 6.172) | |
| ar2 | -0.376 (-0.967 ; 0.215) | 0.213 |  |  |  |  |  |  | |
| ma1 | -0.256 (-1.484 ; 0.972) | 0.683 |  |  |  |  |  |  | |
| sma1 | -0.596 (-0.996 ; -0.195) | 0.004 |  |  |  |  |  |  | |
| Instant trend | -0.047 (-0.123 ; 0.029) | 0.224 |  |  |  |  |  |  | |
| Post change trend | 0.015 (0.003 ; 0.028) | 0.015 |  |  |  |  |  |  | |
| **Autoregressive ARIMA model without differencing (primary outcome)** | | | | | | | | | |
| ar1 | 0.342 (0.143 ; 0.541) | <0.001 | -244.176 | -221.097 | 0.057 | / | / | 6.782 (5.961 ; 7.603) | |
| ar2 | 0.037 (-0.181 ; 0.254) | 0.740 |  |  |  |  |  |  | |
| ar3 | 0.209 (0.003 ; 0.416) | 0.0472 |  |  |  |  |  |  | |
| sar1 | 0.854 (0.758 ; 0.950) | <0.001 |  |  |  |  |  |  | |
| Intercept | 0.562 (0.408 ; 0.717) | <0.001 |  |  |  |  |  |  | |
| Pre change trend | 0.006 (0.003 ; 0.008) | <0.001 |  |  |  |  |  |  | |
| Instant trend | -0.031 (-0.100 ; 0.038) | 0.371 |  |  |  |  |  |  | |
| Post change trend | 0.018 (0.012 ; 0.024) | <0.001 |  |  |  |  |  |  | |

**Appendix II:** Sensitivity analyses of the segmented models used to study monthly consumption of methylphenidate in DDD/TID from January 2016 to December 2023. **Legend**: DDD/TID: Defined daily dose per thousand inhabitants a day; 95%CI: 95% confidence interval; 95%PI: 95% prediction interval; p: statistical value; AIC: Akaike information criterion; BIC: Bayesian Information Criterion; RMSE: root mean square error ; RSQ: R-Squared.

| **Euros per 1,000 inhabitants** | **Trend (95%CI)** | **p** | **AIC** | **BIC** | **RMSE** | **RSQ** | **Adj-RSQ** | **Excess of million euros (95%PI)** |
| --- | --- | --- | --- | --- | --- | --- | --- | --- |
| **Newey-West Standard Error Regression** | | | | | | | | |
| Intercept | 13.520 (12.501 ; 14.539) | <0.001 | 419.001 | 431.823 | / | 0.8 | 0.793 | 166.369 (143.607 ; 189.134) |
| Pre change trend | 0.043 (0.018 ; 0.068) | 0.001 |  |  |  |  |  |  |
| Instant trend | 0.984 (-0.339 ; 2.307) | 0.148 |  |  |  |  |  |  |
| Post change trend | 0.342 (0.271 ; 0.413) | <0.001 |  |  |  |  |  |  |
| **Regression with Month Adjustment** | | | | | | | | |
| Intercept | -1153.982 (-1485.517 ; -822.446) | <0.001 | 310.888 | 351.917 | / | 0.948 | 0.939 | 152.734 (139.826 ; 165.641) |
| Pre change trend | 0.579 (0.415 ; 0.744) | <0.001 |  |  |  |  |  |  |
| Instant trend | 0.223 (-0.795 ; 1.241) | 0.669 |  |  |  |  |  |  |
| Post change trend | 0.361 (0.307 ; 0.414) | <0.001 |  |  |  |  |  |  |
| **Regression with Fourier Transform** | | | | | | | | |
| Intercept | 13.463 (12.720 ; 14.206) | <0.001 | 365.283 | 388.362 | / | 0.895 | 0.886 | 156.847 (139.702 ; 173.990) |
| Pre change trend | 0.046 (0.028 ; 0.065) | <0.001 |  |  |  |  |  |  |
| Instant trend | 0.508 (-0.881 ; 1.897) | 0.475 |  |  |  |  |  |  |
| Post change trend | 0.351 (0.278 ; 0.425) | <0.001 |  |  |  |  |  |  |
| **ARIMA model with seasonal and non-seasonal differencing** | | | | | | | | |
| ar1 | -0.396 (-1.116 ; 0.324) | 0.281 | 269.452 | 286.383 | 0.899 | / | / | 83.343 (52.071 ; 114.560) |
| ar2 | -0.280 (-0.723 ; 0.163) | 0.215 |  |  |  |  |  |  |
| ma1 | -0.299 (-1.086 ; 0.487) | 0.456 |  |  |  |  |  |  |
| sma1 | -1.000 (-1.478 ; -0.522) | <0.001 |  |  |  |  |  |  |
| Instant trend | -0.929 (-2.487 ; 0.629) | 0.242 |  |  |  |  |  |  |
| Post change trend | 0.329 (0.142 ; 0.516) | <0.001 |  |  |  |  |  |  |
| **Autoregressive ARIMA model without differencing (primary outcome)** | | | | | | | | |
| ar1 | 0.401 (0.203 ; 0.599) | <0.001 | 334.072 | 357.152 | 1.164 | / | / | 115.468 (96.763 ; 134.175) |
| ar2 | 0.097 (-0.123 ; 0.316) | 0.388 |  |  |  |  |  |  |
| ar3 | 0.170 (-0.033 ; 0.373) | 0.100 |  |  |  |  |  |  |
| sar1 | 0.828 (0.726 ; 0.930) | <0.001 |  |  |  |  |  |  |
| Intercept | 12.346 (8.704 ; 15.988) | <0.001 |  |  |  |  |  |  |
| Pre change trend | 0.080 (0.016 ; 0.145) | 0.014 |  |  |  |  |  |  |
| Instant trend | -0.682 (-2.181 ; 0.816) | 0.372 |  |  |  |  |  |  |
| Post change trend | 0.329 (0.184 ; 0.474) | <0.001 |  |  |  |  |  |  |

**Appendix III:** Sensitivity analyses of the segmented models used to study monthly expenditure in euros per 1000 inhabitants linked to methylphenidate consumption from January 2016 to December 2023. **Legend**: 95%CI: 95% confidence interval; 95%PI: 95% prediction interval; p: statistical value; AIC: Akaike information criterion; BIC: Bayesian Information Criterion; RMSE: root mean square error ; RSQ: R-Squared.

### **Appendix IV:** Data with year aggregation

| Year | Mean monthly DDD/TID | Euro per 1000 inhabitants | Population (million) |
| --- | --- | --- | --- |
| 2016 | 0.607 | 157.089 | 66.603 |
| 2017 | 0.659 | 175.556 | 66.774 |
| 2018 | 0.720 | 191.908 | 66.992 |
| 2019 | 0.781 | 184.333 | 67.258 |
| 2020 | 0.791 | 177.004 | 67.454 |
| 2021 | 0.951 | 212.261 | 67.626 |
| 2022 | 1.156 | 252.344 | 68.043 |
| 2023 | 1.457 | 314.380 | 68.143 |
